# EUS-assisted preoperative diagnosis of immunoglobulin G4–related cholecystitis mimicking gallbladder cancer in a Mirizzi syndrome case

**DOI:** 10.1097/eus.0000000000000050

**Published:** 2024-02-05

**Authors:** Xiao-Chao Wu, Lin Miao, Su-Min Zhu, Kai-Xuan Wang

**Affiliations:** 1 Medical Centre for Digestive Diseases, The Second Affiliated Hospital of Nanjing Medical University, Nanjing, Jiangsu Province, China; 2 Department of Gastroenterology, Changhai Hospital, Second Military Medical University and Naval Medical University, Shanghai, China.

**Keywords:** Endoscopic ultrasonography, IgG4-related cholecystitis, Gallbladder cancer, Mirizzi syndrome

A 70-year-old woman was transferred to our department with obstructive jaundice, low fever, and right upper abdominal discomfort for 1 week. Contrast-enhanced computed tomography and positron emission tomography/computed tomography revealed Mirizzi syndrome suspected of malignancy. Magnetic resonance cholangiopancreatography revealed type IV Mirizzi syndrome according to Csendes and Beltrán^[1]^ [Figures 1A–C]. As the malignancy remained to be confirmed, EUS was performed and clearly demonstrated Mirizzi syndrome. In detail, the gallbladder wall thickened unevenly and presented sandwich-like structures; huge stones filled the gallbladder cavity without hypoechoic nodule [Figures 2A, B].

**Figure 1:**
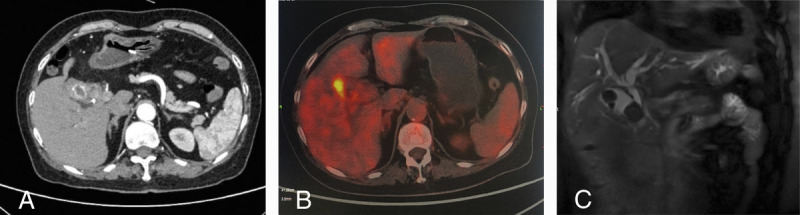
A, Contrast-enhanced CT images revealed gallstones with thickening and strengthening of the gallbladder wall. B, PET/CT detected high-density shadows in the gallbladder, thickened wall of the gallbladder and common hepatic duct (involving adjacent hepatic parenchyma), and a focal hypermetabolic lesion (SUV_max_, 6.1) in and around the area. C, MRCP revealed multiple gallstones and extrinsic compression of the extrahepatic biliary tract, all indicative of Mirizzi syndrome. CT: Computed tomography; MRCP: Magnetic resonance cholangiopancreatography; PET: Positron emission tomography; SUV_max_, Maximum standard uptake value.

**Figure 2:**
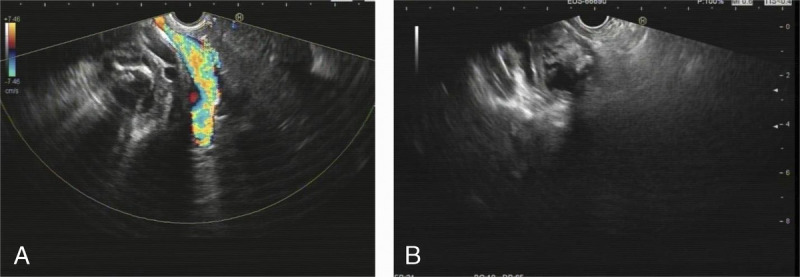
EUS revealed (A) Mirizzi syndrome and huge stones filled the gallbladder cavity. (B) The gallbladder wall thickened unevenly and presented sandwich-like structures.

Subsequent ERCP was applied for temporary drainage of the biliary duct through the nasal passage [Figures 3]. After the condition of the patient was improved, cholecystectomy was performed. Postoperative histopathological examination established a diagnosis of immunoglobulin G4 (IgG4)–related cholecystitis, according to the comprehensive diagnostic criteria for IgG4-related disease revised in 2020^[2]^ [Figures 4A, B].

**Figure 3:**
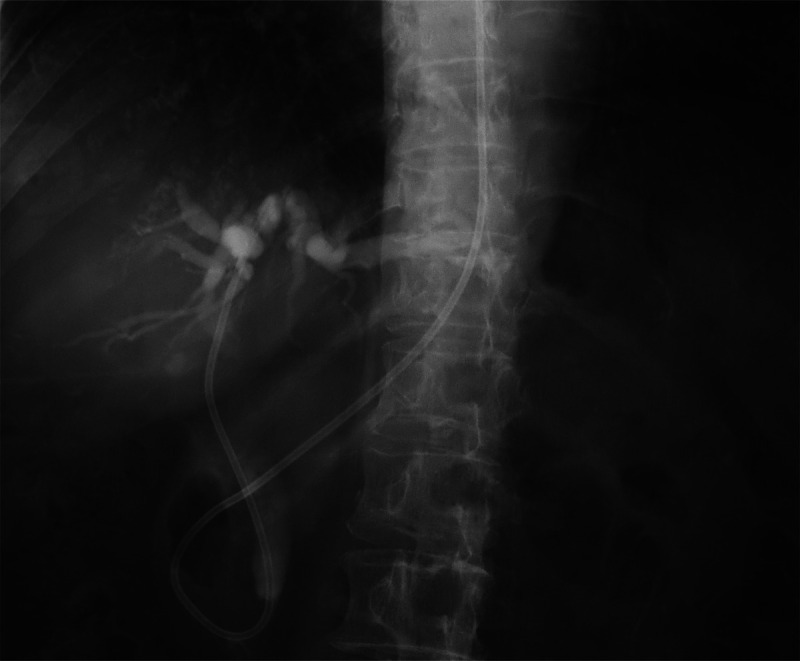
ERCP revealed the common bile duct stenosis. The gallbladder was not visualized. A straight head nasal bile duct was placed for drainage.

**Figure 4:**
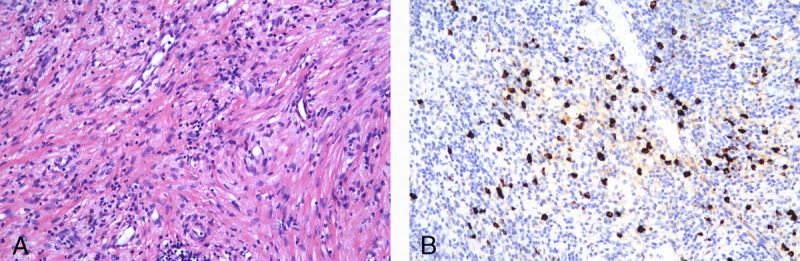
Pathological analysis of the gallbladder bed revealed (A) inflammatory cell infiltration within a background of fibrosis (hematoxylin-eosin stain, original magnification ×200), (B) abundant immunoglobulin G4^+^ (IgG4^+^) plasma cells (immunohistochemistry of IgG4, ×200). IgG4: Immunoglobulin G4.

More than a dozen cases of IgG4-related cholecystitis misdiagnosed as gallbladder cancer have been reported.^[3,4]^ For the first time, we provided a case of using EUS to capture the characteristics of IgG4-related cholecystitis with Mirizzi syndrome as the first symptom. The current case provides a possibility to accurately diagnose IgG4-related cholecystitis using preoperative EUS, which plays a key role. The limitation of this study is that we used to consider whether to perform EUS–fine-needle aspiration for diagnosis, because the gallbladder was blocked by many large stones, whereas the dilated bile duct was prone to cause severe bile leakage, and the patient had the intention to remove the gallbladder, so we gave up EUS–fine-needle aspiration. It could be better visualized by contrast-enhanced EUS. It is our hope that our case can help the reader get inspiration.

## References

[1] Klekowski J Piekarska A Góral M Kozula M Chabowski M. The current approach to the diagnosis and classification of Mirizzi syndrome. *Diagnostics (Basel)* 2021;11(9), 1660. 34574001 10.3390/diagnostics11091660 PMC8465817

[2] Umehara H Okazaki K Kawa S, . The 2020 Revised Comprehensive Diagnostic (RCD) criteria for IgG4-RD. *Mod Rheumatol* 2021;31(3):529–533. 33274670 10.1080/14397595.2020.1859710

[3] Harada Y Mihara K Amemiya R, . Isolated IgG4-related cholecystitis with localized gallbladder wall thickening mimicking gallbladder cancer: a case report and literature review. *BMC Gastroenterol* 2022;22(1):99. 35246051 10.1186/s12876-022-02179-z PMC8895667

[4] Nagai K Kuwatani M Takishin Y, . Immunoglobulin G4–related cholecystitis mimicking gallbladder cancer diagnosed by EUS-guided biopsy. *Endosc Ultrasound* 2022;11(4):334–335. 34494585 10.4103/EUS-D-21-00028 PMC9526093

